# Priorities of the Pediatric Spinal Cord Injury Population: An International Study on Patient-Reported Outcome Measures

**DOI:** 10.3390/children11121415

**Published:** 2024-11-23

**Authors:** Marta Ríos-León, Bashak Onal, Juan Carlos Arango-Lasprilla, Marika Augutis, Allison Graham, Erin Hayes Kelly, Antonis Kontaxakis, Elisa López-Dolado, Anke Scheel-Sailer, Svetlana Valiullina, PEPSCI Collaboration, Julian Taylor

**Affiliations:** 1 Sensorimotor Function Group, Hospital Nacional de Parapléjicos (SESCAM), 45071 Toledo, Spain; juliantaylorgreen2@gmail.com; 2 Instituto de Investigación Sanitaria de Castilla-La Mancha (IDISCAM), 45071 Toledo, Spain; 3 International Spinal Cord Society Paediatric Special Interest Group (ISCoS PaedsSIG), Aylesbury HP21 8AL, UK; bashak.onal@nhs.net (B.O.); marika.augutis@gmail.com (M.A.); allisongrhm@gmail.com (A.G.); a.c.kontaxakis@army.gr (A.K.); elopez@sescam.jccm.es (E.L.-D.); anke.scheel-sailer@paraplegie.ch (A.S.-S.); 4 NHS Buckinghamshire Clinical Commissioning Group, Aylesbury HP20 1UX, UK; 5 Biocruces Bizkaia Health Research Institute, 48903 Barakaldo, Spain; jcarangolasp@vcu.edu; 6 Department of Cell Biology and Histology, University of the Basque Country, 48940 Leioa, Spain; 7 IKERBASQUE, Basque Foundation for Science, 48009 Bilbao, Spain; 8 Department of Neurobiology, Care Sciences and Society, Division of Neurogeriatrics, Karolinska Institutet, 171 77 Stockholm, Sweden; 9 National Spinal Injuries Centre, Buckinghamshire Healthcare NHS Trust, Stoke Mandeville, Aylesbury HP21 8AL, UK; 10 American Academy of Pediatrics, Itasca, IL 60143, USA; ekelly@aap.org; 11 414 Military Hospital for Special Diseases, 152 36 Athens, Greece; 12 Rehabilitation Department, Hospital Nacional de Parapléjicos (SESCAM), 45004 Toledo, Spain; 13 Swiss Paraplegic Centre, 6207 Nottwil, Switzerland; 14 Department of Health Science and Medicine, University of Lucerne, 6002 Lucerne, Switzerland; 15 Clinical and Research Institute of Emergency Pediatric Surgery and Trauma, 119180 Moscow, Russia; vsa64@mail.ru; 16 Harris Manchester College, University of Oxford, Oxford OX1 2JD, UK

**Keywords:** child, adolescent, spinal cord injuries, life and health priorities, rehabilitation

## Abstract

Background/Objectives: Overall priorities of the international pediatric-onset spinal cord injury (SCI) population are unknown. The purpose was to describe and compare Life and Health (L&H) domain overall priorities of European youth with SCI and their parents and caregivers (P&C). Methods: A survey with a cross-sectional design, prepared by the PEPSCI Collaboration, was conducted in six European countries. In total, 202 participants, including youth with SCI (n = 101) and their P&C (n = 101), were included. Overall priorities were calculated based on unhappiness, importance, and research. Results: The sample included youth aged 8–12 years (30.7%) and 13–25 years (69.3%; 38.6% 13–17-year-olds and 30.7% youth aged 18–25 years), in addition to their P&C. The top three L&H priorities highlighted by P&C of the youth aged 8–12 years were “bladder” function (78%), “leg/foot movement” (77%), or “bowel” function (74%), compared with “leg/foot movement” (79%), “sit-to-stand” (76%), or “walking/ability to move” (75%) reported by P&C of the youth aged 13–25 years. The youth aged 13–25 years considered “leg/foot movement” (68%), “bowel” (66%), or “bladder” function (65%) as priorities. The top 10 priorities highlighted by the youth aged 13–25 years compared to the top 10 priorities rated by P&C were issues related to “personal needs”. Nevertheless, “pressure injuries”, “pain”, “bowel function”, or “mobility in the community” were highlighted as top preferences of priorities for the youth aged 13–25 years compared to their P&C. Conclusions: Adolescents/young adults highlighted health domain priorities compared with their P&C, who equally considered L&H domains. Life domains, which were previously unaddressed, were highlighted by P&C, including “adulthood expectations” and “parenthood expectations”. This survey will promote the involvement of stakeholders for comprehensive rehabilitation management for this population.

## 1. Introduction

Pediatric-onset spinal cord injury (SCI) is associated with lifelong issues related to psychosocial and health problems, leading to diminished quality of life and substantial economic costs to the healthcare system [1,2,3]. The European incidence rate is 3.3–6.2 cases per million population per year [2]. The most common causes include motor vehicle accidents (46–74%), spinal tumors (30–63%), autoimmune or inflammatory conditions (28–35%), falls (12–35%), and sports and recreational activities (10–25%) [2].

Compared to adult-onset SCI, pediatric injuries often result in more severe functional impairments due to a higher incidence of complete injuries among children [3]. Additionally, complications related to normal growth (e.g., hip dysplasia, scoliosis) are characteristic issues of pediatric-onset SCI and contribute to functional disabilities [3]. Psychosocial problems (e.g., anxiety/depression), educational challenges, limited social participation, and difficulties with peer integration also significantly impact quality of life [3]. Thus, youth with SCI are impacted by challenges in both life and health (L&H) domains affecting the independence of this population [1]; seeking input from youth and their caregivers regarding prioritization of these needs would help guide the development of a comprehensive approach for rehabilitation programs. 

The needs of individuals with SCI and caregivers are increasingly considered to ameliorate management in this population [4]. As comprehensive pediatric SCI care is family-centered [3], it is essential for parents/caregivers (P&C) to be involved with rehabilitation and setting priorities for these patients. These priorities must be adapted to ensure that new results are considered for improvements in rehabilitation outcomes [5,6]. Research priorities have been previously rated by the Pan-European Pediatric Spinal Cord Injury (PEPSCI) Collaboration as the first part of a larger international study [1], which constitutes the first part of this study. Nevertheless, there is a lack of evidence on overall priorities, including importance and unhappiness priorities, which we aimed to address through this second study of the same population. In this study, we will focus on non-research priorities, such as unhappiness and importance priorities, that constitute part of the overall priorities completely differing from unique research priorities. Furthermore, no research to date has presented an integrated and comprehensive view of the overall priorities, including around the health, social, and emotional status, of youth with SCI and their P&C using an international sample. Thus, this study aims to comprehensively investigate the overall L&H priorities of young adults, adolescents, and children with SCI and their P&C across Europe.

## 2. Materials and Methods

A large cross-sectional survey was performed with European people with SCI according to the Declaration of Helsinki [7]. The study was approved by the Local Ethics Committee at each site (England, Spain, Greece, Switzerland, Russia, and Sweden). Hospital databases and local announcements were used for screening (March 2017–April 2021). An informed consent form was signed by all participants prior to their inclusion in this study [1]. This study constitutes the second part of the PEPSCI study [1] concerning the same population, as previously described.

The eligibility criteria for inclusion were as follows: (1) individuals aged 8 to 25 years with diagnosis of pediatric-onset SCI; (2) SCI resulting from congenital (e.g., Arnold–Chiari malformation, tethered cord syndrome, skeletal malformations) or acquired etiologies (excluding spina bifida); and (3) inpatients and outpatients who had been discharged from acute SCI treatment for at least six months. Exclusion criteria included severe neurological conditions that impact cognitive function (e.g., due to acquired brain injury or encephalocele) affecting patients and P&C [1].

### 2.1. Survey

The survey consists of 3 sections: the *Life and Health Domain Questionnaire (L&HDQ)* (*Part II*, survey), the *Basic Information Form (Part I)*, and the *Neurological Form (Part III)* (Table 1). Data from individuals with pediatric-onset SCI aged 18–25 years were specifically analyzed to assess the priorities of adolescents transitioning into young adulthood [1].

The *L&HDQ* (Supplementary Materials) was developed by the PEPSCI Collaboration [1] on the basis of results from a systematic review [5], which describes L&H priorities considering the International Classification of Functioning, Disability, and Health (ICF) domains in adults with SCI. This survey evaluated L&H domains based on happiness, importance, and research priorities (Table 1). A 5-point Likert scale, ranging from 1 (“very unimportant”/“very unhappy”) to 5 (“very important”/“very happy”), was used to rate responses for each item. For the analysis, the happiness scores were reversed: 1 symbolized “very happy” and 5 represented “very unhappy” [1].

The *L&HDQ* was translated into Greek [1], Spanish [1], German [1], Russian [1], and Swedish [1] following established guidelines [8] and a two-step cross-cultural adaptation process [8,9]. The versions of the *L&HDQ* for youth aged 8–12 years and those aged 13–25 years and P&C were completed by children (8–12 years), adolescents/young adults (13–25 years), and all P&C, respectively (Table 1) [1].

### 2.2. Data Analysis

The responses were reviewed for clarity and completeness. Data analysis focused on individuals with SCI and their P&C. Data analysis was performed using SPSS version 22.0 (IBM, New York, NY, USA), in addition to Sigma Plot version 12.0 (Systat Software, San Jose, CA, USA). The Microsoft Excel 2016 software package (Microsoft, Redmond, WA, USA) was used for descriptive analysis. Percentages, frequencies, medians with 25th and 75th percentiles, and means and SD were analyzed to determine respondents´ responses and characteristics. Percentage and frequency data were analyzed to characterize the neurological and sociodemographic features of people with SCI. For each item of the *L&HDQ*, percentages and medians with 25th and 75th percentiles were analyzed. Scores indicating the highest importance, unhappiness, and research priorities were assessed using a Likert scale (values of “4” and “5”, or “5”) and presented as medians with interquartile range (25th and 75th percentiles) and/or percentages. For each L&H domain item, an overall percentage score was computed from individual unhappiness, importance, and research priority scores. Thus, overall priorities were calculated from *L&HDQ* responses in 13–25-year-olds and all P&C (P&C of 8–12-year-olds and P&C of 13–25-year-olds). The highest overall percentage value was considered to rank the items. Data from the *L&HDQ* were analyzed without interpolating missing data (Table 2). Furthermore, a stratified analysis was conducted based on the severity (motor complete (AIS A-B)/incomplete (AIS C-D) injury), SCI functional level (tetraplegia/paraplegia) [1,10], time since injury (less/more than 5 years), and sex (man/woman). Comparisons of stratified data, categorized into non-important (values of “3” or lower) and important (values of “4” and “5”), were assessed using Pearson’s chi-squared test or Fisher´s exact test. A *p*-value of <0.05 was considered statistically significant (95% confidence level).

## 3. Results

A total of two hundred and two subjects filled out the surveys (101 completed by 8–25-year-olds with SCI (58.4% men; age, 15.6 ± 4.3 years; Table 2) [96% response rate] and their P&C [98% response rate, 101 dyads]). In accordance with the International Spinal Cord Injury Core Data Set (Version 2.0) [11], standardized data reporting is presented in Table 2, which also reflects the age groups initially established by the PEPSCI Collaboration for two parts of the survey (*Part II and I*).

**Figure 1:**
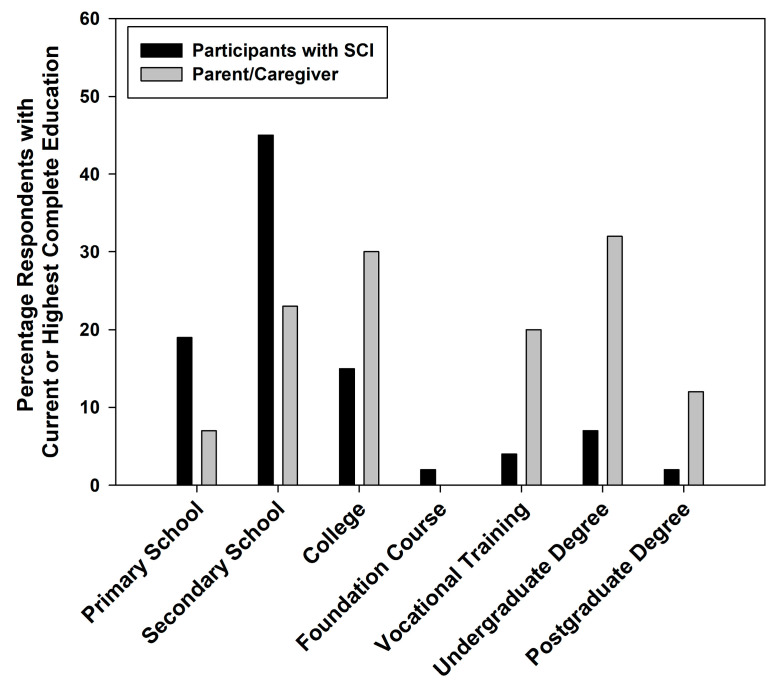
Educational levels of individuals with SCI along with those of their P&C.

Among young adults, children, and adolescents with SCI, the majority were diagnosed with motor incomplete SCI (55.4%) with traumatic etiology (51.5%), levels of neurological injury ranging from T1 to S5 (62.3%), and duration of injury less than 5 years of evolution (53.5%) (Table 2). Additionally, the majority of young individuals with SCI presented paraplegia (61.3% children; Table 2). The leading traumatic causes of SCI related to traffic and land transport, followed by falls and sports-related accidents (75%). Figure 1 presents an overview of the educational levels of individuals aged 8–25 years and their P&C.

### 3.1. Individuals Aged 8–12 Years

The top ten overall priorities for L&H domains of youth with SCI aged 8–12 years, highlighted by their P&C, are presented in Table 3. The top three overall health priorities were “bladder” function, “leg/foot movement”, and “bowel” function. “General health/feel”, “physical function”, and “assistive technologies” were highlighted as the top three overall life priorities.

“Pressure injuries”, “sit-to-stand”, and “bowel” function were considered the top three L&H domain unhappiness priorities (Supplementary Table S4).

### 3.2. Individuals Aged 13–25 Years

The top ten overall priorities for L&H domains of 13–25-year-olds, highlighted by individuals with SCI and their P&C, are presented in Table 4. For adolescents and young adults, “leg/foot movement”, “bowel”, or “bladder” function were considered the top three overall health priorities, while “mobility in the community”, “physical function”, and “fitness/exercise” were highlighted as the top three overall life priorities. In fact, “mobility in the community”, “sit-to-stand”, and “fitness/exercise” were highlighted as the main three L&H domain unhappiness priorities (Supplementary Table S5). For their P&C, “leg/foot movement”, “sit-to-stand”, and “walking/ability to move” were considered the top three overall health priorities; however, “physical function”, “fitness/exercise”, or “general health/feel” were highlighted as the top three overall life priorities (Table 4). Table 4 *(see footnotes)* also presents the overall priorities for individuals aged 13–17 years and those aged 18–25 years, as reported by subjects with SCI and their P&C.

### 3.3. Comparisons of Overall Priorities According to Groups and Age Ranges

**Figure 2:**
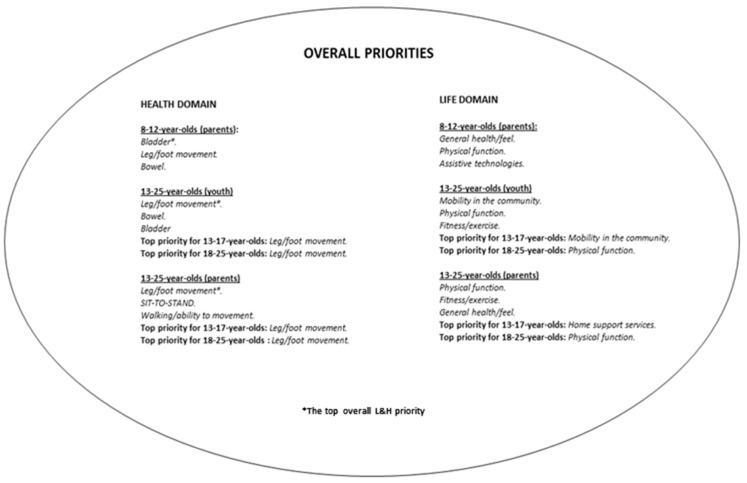
Summary of the top 3 overall priorities for life and health domains highlighted by individuals with SCI and their P&C. Regarding both life and health domains, the asterisk symbolizes the top overall priority for children (8–12 years), young adults, and adolescents (13–25 years) considered by their P&C, and the top priority (overall) highlighted by adolescents/young adults themselves. For both life and health domains, the top ten priorities unaddressed by *Simpson* et al. *(2012)* [5] are highlighted in upper case.

Figure 2 presents a comparison of the top priorities (overall) for groups and age ranges. Similar top priorities (overall) for L&H domains of individuals with SCI aged 8–12 years and those aged 13–25 years, as rated by P&C, are shown (Table 3 and Table 4). In addition, the P&C of children (Table 3) and adolescents/young adults (Table 4) highlighted similar scores for “physical function” (72% vs. 71%), “general mood” (63% vs. 64%), “parenthood expectations” (62% vs. 63%), and “leg/foot movement” (77% vs. 79%) priorities. For individuals aged 13–25 years, the top 10 priorities (overall) rated by P&C and those reported by adolescents/young adults themselves also demonstrated similarities, particularly in relation to scores for “personal needs” (62% vs. 61%; Table 4). In fact, the top ten L&H domain overall priorities preferred for adolescents/young adults, compared with the top ten priorities rated by their P&C, were issues associated with “personal needs” (Table 5).

In general, regarding comparisons across groups and age ranges, unique overall priorities were highlighted. The 13–25-year-olds highlighted unique top ten priorities as “mobility in the community”, “ease of arrival to destination”, “mobility at place of education”, “employment expectations”, and “bowel” function, which were not considered top ten priorities by their P&C (Table 4 and Table 5). Their P&C rated “sexual expectations” and “concentration/learning” unique top ten priorities; however, these priorities were not highlighted as top ten priorities by adolescents/young adults (Table 4 and Table 5) or P&C of individuals aged 8–12 years (Table 3). P&C of children reported “adulthood expectations”, “dating expectations”, “spasms”, “pain”, and “pressure injuries” as top priorities (Table 3); however, these priorities were not highlighted as top ten priorities (overall) by young adults/adolescents (Table 4).

### 3.4. Differences in L&HDQ Items Considering the Severity, Functional Level or Duration of Injury, and Sex

Regarding overall priority items, significant differences were found based on sex, severity, and time since injury in 13–25-year-olds. Girls showed significantly higher overall priorities scores than boys in “mobility at place of education”, “dressing/undressing”, “leg/foot movement”, “sit-to-stand”, “transfer movements”, and “bladder” and “bowel” function items (*p* < 0.05). Nevertheless, no significantly higher overall priorities scores were found in boys when compared with those in girls (*p* > 0.05).

Participants with motor complete SCI rated higher overall priorities scores than individuals with motor incomplete injury in “assistive technologies” (*p* = 0.04). Additionally, individuals with SCI less than 5 years after injury showed the highest overall priorities scores in the “leg/foot movement” item (*p* = 0.03).

Adolescents and young adults with paraplegia and those with tetraplegia reported no significant differences in overall priorities scores (*p* > 0.05). Nevertheless, 13–25-year-olds with tetraplegia reported significantly greater unhappiness related to the “personal needs” and “transfer movements” items compared to youth with paraplegia (*p* < 0.05; Supplementary Table S5). Significant differences in L&HDQ items related to unhappiness priorities for children were also found (*see* Supplementary Table S4 for details).

## 4. Discussion

This study provides the first comprehensive overview of overall priorities for L&H domains reported by young adults, children, and adolescents with SCI, in addition to their P&C, in Europe. For P&C, the top priorities differed by age group: “bladder” function was the top priority (overall) for 8–12-year-olds, while “leg/foot movement” was the top priority (overall) for 13–25-year-olds. Among the 13–25-year-olds themselves, “leg/foot movement” was considered the top overall priority, with “mobility in the community” emphasized as their top unhappiness priority.

These findings align with several L&H priorities identified by subjects with adult-onset SCI, as reported in previous studies [5,6,12,13,14,15,16]. Variations in L&H priorities between young adults, children, and adolescents and their P&C might reflect evolving needs during transitions from adolescence to young adulthood or from childhood to adolescence. Indeed, developmental factors could influence these priorities by impacting on various areas, such as mobility, bladder or bowel function, and complications management [17]. Many of the priorities identified are consistent with developmental goals for young individuals [18] and emphasize the importance of factors associated with access to healthcare or general health, in addition to mobility. Furthermore, these priorities resemble those found in children with neurodevelopmental disorders, such as spina bifida, focusing on daily care, mobility, and socialization [18]. Nevertheless, similarly to adults SCI priorities [12,13], adolescents and young adults emphasized bladder, bowel, or motor functions, such as foot or leg movement. These results highlight the need to address developmental factors or specific age-related needs to enhance the management of individuals with pediatric-onset SCI.

### 4.1. Life Domain Priorities

The top priorities (overall) for the life domain considered by 13–25-year-olds and the P&C of youth with SCI aged 8–25 years were similar to adults SCI priorities [5,19]. Young adults or adolescents with SCI, and all P&C, reported physical function, fitness/exercise, home support, and assistive technologies as the most important top life priorities (overall), which are similar to those considered by adults [5]. These results highlight the relevance of physical status influencing life satisfaction and quality of life in this population [20]. Furthermore, adolescents and young adults highlighted ease of arrival to destination, employment expectations, and mobility at place of education within the top 10 overall priorities, unlike the top 10 research priorities previously reported by adolescents and young adults with SCI [1]. The reported mobility needs, particularly highlighted by girls due to the importance of independence and self-management for them [21], may be associated with certain issues, such as inadequate accessibility to public spaces and buildings or lack of suitable mobility aids, which are significant environmental barriers for individuals with SCI [22]. Thus, assistive technology, which could improve independence, is particularly highlighted by children with motor complete injury due to their important restrictions in mobility. Additionally, all P&C highlighted general mood and general health/feel as important overall life priorities due to the association between clinical factors, such as short duration of injury, and anxious states and poor social relationships [23].

The current study also highlighted certain areas, such as parenthood or adulthood expectations, that were not previously included in the top 25% of adults SCI priorities [5]. The new unreported areas may be linked to emotional or social challenges in children with SCI, such as minor opportunities for peer interaction [24]. Thus, these new life priorities might be associated with the adaptation process in stages of adolescence and should be highlighted as points of intervention for a comprehensive pediatric rehabilitation program.

Additionally, young adults or adolescents and their P&C highlighted personal needs as an important overall life priority. In fact, young adults or adolescents with paraplegia reported significantly lower levels of unhappiness compared to those with tetraplegia. These results suggest that limited mobility, particularly for individuals with tetraplegia, has a substantially negative impact on psychosocial function. Tetraplegia has been previously considered as a major factor related to limitations in activity and participation, in addition to lower quality of life, in the SCI population [25]. Additionally, tetraplegia in adults has been associated with lower levels of life satisfaction when compared to paraplegia [26].The greater physical challenges associated with tetraplegia may contribute to these findings.

The relevance of addressing specific life domain items, in addition to the need for the development of new assessment tools to identify priorities previously unaddressed (e.g., the Child Needs Assessment Checklist [27] or others [28]), have been highlighted by the current findings. Furthermore, enhancing these measurement tools will be crucial for improving rehabilitation management within a psychosocial framework for this population.

### 4.2. Health Domain Priorities

Regarding health priorities (overall), the young adults or adolescents and P&C of youth with SCI aged 8–25 years highlighted similar priorities compared to adults with SCI [5,12,13,14,15,16,19]. The most common and relevant overall health priorities were lower extremity and bladder functions, mobility (including walking, transfer movements, or sit-to-stand abilities), and respiratory functions (including breathing or coughing). These priorities reflect those commonly emphasized by adults with SCI [5,12,13,14,15,16]. Furthermore, adolescents and young adults highlighted transfer movements, eating/drinking, and dressing/undressing within the top 10 overall priorities, unlike the top 10 research priorities previously reported by adolescents and young adults with SCI [1]. Additionally, both young adults or adolescents and their P&C also prioritized arm or hand function, a concern shared with adult SCI populations [5,13,14,15,16,19]. These findings support the consideration of upper extremity impairments as important complications in children with tetraplegia related to limited mobility and access [25].

Several heath priorities have previously been emphasized as key goals for rehabilitation in the adult SCI population (e.g., walking, transfer movements, bladder function, or hand and arm function) [4,12]. Importantly, rehabilitation of hand or arm function is particularly important for enabling young people to carry out self-care tasks and daily living activities, including bladder management via intermittent urinary self-catheterization [29], as urinary incontinence may be present in more than 70% of children with SCI [30]. Indeed, recommendations for the pediatric SCI population include teaching self-catheterization to youth older than 5 years and addressing pressure injury prevention and care [17,29]. These findings highlight the relevance of prioritizing these areas in pediatric SCI care, an approach supported by previous research [29]. Furthermore, this research brought attention to priorities, e.g., sit-to-stand function (Figure 2), that were not highlighted in the top 25% adult SCI priorities [5]. As the recovery of sit-to-stand function is critical for functional gait rehabilitation, incorporating activity-based therapies, such as locomotor training [31], could enhance management in youth with SCI.

This study also confirmed that both girls and individuals with short durations of injury particularly highlighted leg/foot movement, suggesting the importance of considering individual and clinical characteristics to manage pediatric SCI. Furthermore, transfer movements, an overall priority highlighted by girls, was also highlighted as an area of unhappiness by adolescents/young adults with tetraplegia. The physical challenges experienced by individuals with paraplegia are less severe than those faced by those with tetraplegia, which might account for these findings. Furthermore, sex differences observed in the adult SCI population [31] highlight the necessity of research focused on female-specific issues to improve personalized care.

The relevance of this research focused on offering a potential framework for addressing health and psychosocial priorities across various age groups of youth with SCI with the aim of enhancing their management and support. Rehabilitation programs should integrate the priorities of youth and their P&C for a patient-centered (individual priority needs [27]) and goal-oriented approach from a multidisciplinary and preventive perspective.

### 4.3. Limitations

Several limitations should be considered. First, in addition to developmental factors that might influence the differences in reported priorities across age groups, the reported L&H priorities could also be affected by how children accept their circumstances, which influences their life satisfaction and participation [24]. This could explain variations in reported priorities for L&H domains between subjects with pediatric-onset SCI and P&C. Nevertheless, both youth and P&C´s needs, which may be different, are important and must be considered and included. Second, the study addressed only broad life and health areas. Future research should develop an in-depth investigation into the impact of each area of priority and evaluate the effectiveness of targeted therapeutic approaches based on these areas. Additionally, this study underscores the need for further research using a biopsychosocial framework from a multidisciplinary and interdisciplinary approach for the management of individuals with pediatric-onset SCI. The development of international guidelines for systematically monitoring psychosocial and health status, including the use of additional tools (e.g., telerehabilitation), should be kept in mind. Further international research is essential to advance these areas for pediatric SCI populations.

## 5. Conclusions

Young adults or adolescents with SCI underscored overall priorities for health domains (e.g., leg/foot and arm/hand movement, bowel/bladder function, transfer movements, sit-to-stand). Leg/foot movement and assistive technologies were the unique priority items showing significant differences in duration of injury and injury severity, respectively. Girls highlighted sit-to-stand, a priority not previously reported. Young adults or adolescents and all P&C highlighted health domains as the top L&H priorities (overall), while P&C underscored life domains (e.g., parenthood or adulthood expectations that were not originally highlighted) [5]. Thus, these findings encourage rehabilitation professionals to collaborate closely with young people with SCI and their P&C to address L&H priorities that have been overlooked in previous studies of the adult SCI population. In addition, rehabilitation professionals should focus on age-specific needs to promote a multidisciplinary and comprehensive care plan to effectively meet these priorities.

## Figures and Tables

**Table 1:** Overview of survey components.

**Basic Information Form: Part I**
*The Basic Information Form (see* File S1) consisted of twelve items associated with clinical and demographic data of individuals with SCI. Participants with pediatric-onset SCI aged 18–25 years (self-report questionnaire) or P&C of those aged 8–17 years filled out this form [1].
**L&HDQ: Part II**
The *L&HDQ* evaluated L&H domains, which were rated based on importance (“how important each item is in the daily life of the child”), happiness (“how happy or unhappy the child was with this part of his/her life”), and research priorities (“how important or unimportant the SCI problem is for the participant’s doctor to study”). For each response (item), a 5-point Likert scale was used, where 1 indicated “very unimportant” or “very unhappy” and 5 denoted “very important” or “very happy”. A free-text option was also provided to add any priorities related to SCI not included in the questionnaire [1]. For children with SCI **(8–12 years)**, the *L&HDQ* consisted of 7 items for research priorities (all concerning life domains) and 36 items for unhappiness priorities (22 and 14 related to life and health domains, respectively). This version was developed by the PEPSCI expert committee to address common concerns of children [1,3]. Assistance from P&C was available to clarify any questions for children aged 8–12 years, who completed the questionnaire [1]. For young adults and adolescents with SCI (**13–25 years**), the *L&HDQ* comprised 45 items (17 and 28 related to health and life domains, respectively) for unhappiness, importance, and research priorities [1]. Support from the research team and the leading clinician for clarification or assistance was provided as needed [1]. For **P&C,** the *L&HDQ* included forty-four items (17 and 27 health and life domains, respectively), which rated unhappiness, importance, and research priorities for individuals aged 8–25 years with SCI by their P&C. Its structure or format were similar to the version for people aged 13–25 years; however, the life domain item “How you look” (L5) was excluded for P&C [1]. The *L&HDQ* was translated into Greek, Swedish, Spanish, Russian, and German following established guidelines [8,9] and a two-step cross-cultural adaptation process [1,8,9]. Firstly, two bilingual translators from each country (native Greek, Spanish, Swedish, German, and Russian speakers), who were experienced in SCI rehabilitation and colloquial pediatric language, translated the questionnaire from English into Greek, Spanish, Russian, German, and Swedish [1]. Secondly, an expert committee of 4 translators in each country, including 2 researchers, reviewed and assessed 2 independent translations for content equivalences and conceptual and semantic criteria in each language [1].
**Neurology Form: Part III**
*The Neurology Form* (*see* File S1) was designed to collect basic information associated with the clinical characteristics of SCI. This form was completed by the healthcare professional [1]. The severity of SCI was assessed using the American Spinal Injury Association Impairment Scale (AIS) in accordance with the International Standards for Neurological Classification of Spinal Cord Injury (ISNCSCI) examination. The AIS and ISNCSCI were utilized to determine the severity and sensory and motor impairment of a SCI (refer to *Neurology form, Part III*). Motor incomplete was classified as AIS C-D, while motor complete SCI was classified as AIS A-B [1,10].

*Note*: All participants filled out *Part II* and *Part I* either at the hospital, under the guidance of the research team or lead clinicians to ensure accuracy and a high response rate, or at home. For those completing the survey at home, the research team or lead clinicians contacted participants by phone to address incomplete or unclear responses, thus ensuring complete data collection. Healthcare professionals filled out the *Neurology Form (Part III).* Standardized written instructions were provided for completing the 3 parts of the surveys across all age groups (i.e., children (8–12 years), adolescents and young adults (13–25 years), and P&C of participants aged 8–25 years).

**Table 2:** SCI and demographic characteristics of 8–25-year-olds with pediatric-onset SCI.

Variables	N (%)
**Age groups (years)** ^¥^	
8–25	101 (100)
***6–12***	31 (30.7)
8–12	31 (30.7)
13–25	70 (69.3)
13–17	39 (38.6)
***13–14***	6 (5.9)
***15–17***	33 (32.7)
18–25	31 (30.7)
***18–21***	23 (22.8)
Sex *	
Men	59 (58.4)
Women	42 (41.6)
**Neurological level ^a^, severity ^b^, and functional grade ^c^ of injury**	
C1-4 ASIA Impairment Scale (AIS) A-B or C-D	5 (5.0)/10 (9.9)
C5-8 ASIA Impairment Scale grade (AIS) A-B or C-D	5 (5.0)/7 (6.9)
T1-S5 Impairment Scale grade (AIS) A-B or C-D	27 (26.7)/36 (35.6)
Paraplegia/tetraplegia ^†^/Cauda equina	62 (61.4)/32 (31.7)/5 (5.0)
Motor incomplete/complete ^d,‡^	56 (55.4)/38 (37.6)
**Duration of injury (years) ^e^**	
0–5 ^§^	54 (53.5)
≥5	43 (42.6)
**Etiology ^f^**	
Non-traumatic/traumatic	38 (37.6)/52 (51.5)
Congenital ^g^/Other—not specified	4 (4.0)/4 (4.0)

*Note:* Age groups followed the standards recommended by the *International Spinal Cord Injury Core Data Set (Version 2.0,* bold and italics data), as defined in *Part II* and *Part I* of the survey (underlined data, *see* Supplementary Materials). Missing data are indicated by lower-case superscript letters. AIS: American Spinal Injury Association Injury Severity. ASIA: American Spinal Injury Association. N: number. ^a^ n = 11 missing data. ^b^ n = 2 missing data. ^c^ n = 9 missing data. ^d^ n = 7 missing data. ^e^ n = 4 missing data. ^f^ n = 3 missing data. ^g^ Congenital pathologies are categorized as “other congenital skeletal malformations [specific etiology not reported]” (n = 3) and “congenital syringomyelia” (n = 1). The non-traumatic SCI etiologies are classified as “inflammation/infection” (n = 8), “transverse myelitis” (n = 12), “tumor” (n = 13), and “vascular disorders” (n = 5). The traumatic SCI etiologies are categorized as “motor vehicle/pedestrian accident” (n = 17), “surgical complication” (n = 6), “birth trauma/injury” (n = 3), “other accident” (n = 4), “sports” (n = 11), and “fall” (n = 11). ^¥^ Distribution of the sample according to country: *Spain* (n = 31): 12 children (8–12-year-olds), 19 adolescents and young adults (13–25-year-olds); *England* (n = 26): 9 children (8–12-year-olds), 17 adolescents and young adults (13–25-year-olds); *Russia* (n = 15): 4 children (8–12-year-olds), 11 adolescents and young adults (13–25-year-olds); *Switzerland* (n = 13): 3 children (8–12-year-olds), 10 adolescents and young adults (13–25-year-olds); *Sweden* (n = 11): 2 children (8–12-year-olds), 9 adolescents and young adults (13–25-year-olds); *Greece* (n = 5): 1 child (8–12-year-olds), 4 adolescents and young adults (13–25-year-olds). * Men: 45.2% of individuals aged 8–12 years (n = 14), 64.3% of those aged 13–25 years (n = 45); women: 54.8% of those aged 8–12 years (n = 17), 35.7% of those aged 13–25 years (n = 25). ^†^ Tetraplegia: 25.8% of individuals aged 8–12 years, 34.3% of those aged 13–25 years; paraplegia: 61.3% of those aged 8–12 years, 60% of those aged 13–25 years. ^‡^ Motor complete (AIS A-B): 33.3% of individuals aged 8–12 years, 40% of those aged 13–25 years; motor incomplete (AIS C-D): 63.3% of those aged 8–12 years, 52.9% of those aged 13–25 years. ^§^ Six months was the shortest duration of injury. *It is important to note that researchers contacted participants and/or clinicians to complete uncompleted data. If no response was received after more than three attempts or there was restricted access to some missing data, the data were considered missing.*

**Table 3:** The top ten overall, unhappiness, research, and importance priorities for life and health domains highlighted by P&C of 8–12-year-olds with SCI (n = 31), according to the percentage of “very important” (5) and “important” (4) scores.

	Overall *	Importance	Unhappiness	Research
**Life Domains**				
*General health/feel (L1)*	73 ^†^	93	37	89
*Physical function (L2)*	71	92	33	88
*Assistive technologies (L19)*	68	96	19	83
*Employment expectations (L23)*	66	96	22	76
*Adulthood expectations (L27)*	66	89	27	76
*General mood (L3)*	64	96	8	84
*Dating expectations (L24)*	63	96	24	63
*Fitness/exercise (L4)*	63	89	25	72
*Parenthood expectations (L26)*	62	84	21	72
*Home Support Services (L18)*	62	91	20	68
**Health Domains**				
*Bladder (H11)*	78 ^‡^	100	40	91
*Leg/foot movement (H7)*	77 ^‡^	92	48	88
*Bowel (H12)*	74 ^‡^	100	32	86
*Spasms (H16)*	74 ^‡^	89	44	83
*Walking/ability to move (H9)*	71 ^‡^	95	27	91
*Pain (H15)*	70	96	22	88
*Pressure injuries (H17)*	69	90	29	82
*Sit-to-stand (H8)*	66	88	19	86
*Transfer movements (H10)*	63	100	6	77
*Breathing/coughing (H3)*	61	90	17	71

*Note:* * The percentage value (overall) of very important and important scores was used for ranking the L&H priorities. ^†^ Response rate: 100%. ^‡^ Response rate: <70%.

**Table 4:** The top ten overall, unhappiness, research, and importance priorities for life and health domains highlighted by 13–25-year-olds with SCI and their P&C (n = 70), according to the percentage of “very important” (5) and “important” (4) scores.

13–25-Year-Olds with SCI					P&C of 13–25-Year-Olds with SCI				
	Overall *	Importance	Unhappiness	Research		Overall *	Importance	Unhappiness	Research
**Life Domains**									
*Mobility in the community (L15)*	63	94	28	68	*Physical function (L2)*	72 ^†^	94	29	93
*Physical function (L2)*	63 ^†^	86	23	80	*Fitness/exercise (L4)*	68 ^†^	99	21	83
*Fitness/exercise (L4)*	62	88	25	75	*General health/feel (L1)*	67 ^†^	96	14	90
*Personal needs (L18)*	61	97	21	66	*Home Support Services (L18)*	65	85	33	65
*Healthcare access (L16)*	60	94	9	78	*Assistive technologies (L19)*	65	95	23	76
*Assistive technologies (L20)*	58 ^‡^	93	12	70	*Healthcare access (L15)*	64	97	15	79
*Ease of arrival to destination (L17)*	57	92	21	60	*Parenthood expectations (L26)*	63	85	26	78
*Employment expectations (L23)*	56	96	13	58	*General mood (L3)*	63	97	9	84
*Mobility at place of education (L22)*	55	90	15	59	*Personal needs (L17)*	62 ^‡^	100	14	71
*Home Support Services (L19)*	55 ^‡^	85	15	64	*Sexual expectations (L25)*	62	88	24	70
**Health Domains**									
*Leg/foot movement (H7)*	68 ^‡^	92	26	84	*Leg/foot movement (H7)*	79 ^‡^	93	56	88
*Bowel (H12)*	66 ^‡^	94	19	85	*Sit-to-stand (H8)*	76 ^‡^	93	54	83
*Bladder (H11)*	65	94	17	84	*Walking/ability to move (H9)*	75	89	48	89
*Sit-to-stand (H8)*	64	87	26	78	*Arm/hand movement (H4)*	73 ^‡^	98	37	83
*Transfer movements (H10)*	64	98	20	74	*Eating/drinking (H5)*	71 ^‡^	98	38	77
*Arm/hand movement (H4)*	64	98	13	78	*Dressing/undressing (H6)*	71 ^‡^	96	38	75
*Walking/ability to move (H9)*	63	95	17	78	*Breathing/coughing (H3)*	70	96	30	82
*Eating/drinking (H5)*	62	97	13	75	*Bladder (H11)*	69	89	34	88
*Dressing/undressing (H6)*	62	98	15	71	*Transfer movements (H10)*	68 ^‡^	90	35	78
*Breathing/coughing (H3)*	61	92	8	81	*Concentration/learning (H1)*	67	97	31	75

*Note:* The top ten L&H domain overall priorities for 13–25-year-olds with SCI or P&C of 13–25-year-olds are indicated with grey shading. SCI: spinal cord injury. * The percentage value (overall) of very important and important scores was used for ranking the L&H priorities. ^†^ Response rate: 100%. ^‡^ Response rate: <70%. The overall priorities for individuals aged 13–17 years and 18–25 years, as reported by young adults or adolescents with SCI and their P&C, were analyzed, too. Adolescents (13–17 years) considered “leg/foot movement” (66%), “walking/ability to move” (64%), and “bowel” function (63%) as the top 3 overall priorities for L&H domains, while their P&C rated “leg/foot movement” (79%), “walking/ability to move” (77%), and “bladder” function (74%) as the top 3 priorities (overall) for L&H domains. The young adults (18–25 years) highlighted “leg/foot movement” (71%), “bladder” function (70%), and “bowel” function (69%) as the top 3 overall priorities for L&H domains; however, P&C reported “leg/foot movement” (76%), “breathing/coughing” (76%), and “sit-to-stand” (75%) as top 3 priorities (overall) for L&H domains.

**Table 5:** Overall priorities for life and health domains preferred by 13–25-year-olds with SCI (n = 70) when compared to P&C of 13–25-year-olds (n = 70) based on the percentage of “very important” (5) and “important” (4) scores.

13–25-Year-Olds with SCI					P&C of 13–25-Year-Olds with SCI				
	Ratio Youth/P&C % 4 & 5 Scores *	Youth % 4 & 5 Scores ^†^	P&C % 4 & 5 Scores ^†^	Mean Youth—P&C % 4 & 5 Scores ^†^		Ratio Youth/P&C % 4 & 5 Scores *	Youth % 4 & 5 Scores ^†^	P&C % 4 & 5 Scores ^†^	Mean Youth—P&C % 4 & 5 Scores ^†^
**Life Domains**									
*Mobility in the community (L15)*	**103.** **7**	**63.** **4 (#1)**	61.1 (#12)	**62.** **2 (#3)**	*Mobility at home (L14)*	88.0	53.0 (#13)	60.2 (#13)	56.6 (#12)
*Mobility at place of education (L22)*	**103.** **6**	**55.0 (#9)**	53.1 (#23)	54.1 (#16)	*Physical function (L2)*	87.7	**63.0 (#2)**	**71.** **8 (#1)**	**67.** **4 (#1)**
*Mobility at place of education (L22)*	99.7	**57.4 (#7)**	57.6 (#16)	57.5 (#11)	*Relationships place education (L23)*	86.5	46.1 (#24)	53.3 (#22)	49.7 (#24)
*Personal needs (L18)*	98.4	**60.9 (#4)**	**91.** **9 (#9)**	**61.** **4 (#6)**	*General mood (L3)*	86.1	54.4 (#11)	**63.** **2 (#8)**	**58.** **8 (#9)**
*Ability to help others (L10)*	94.5	46.6 (#23)	49.3 (#25)	47.9 (#25)	*Fun and pastimes (L6)*	84.9	48.8 (#19)	57.4 (#17)	53.1 (#19)
*Healthcare access (L16)*	94.3	**60.1 (#5)**	**63.** **7 (#6)**	**61.** **9 (#4)**	*Communication with others (L9)*	84.7	48.5 (#20)	57.3 (#18)	52.9 (#20)
*Employment expectations (L24)*	93.6	**56.2 (#8)**	60.0 (#14)	**58.** **1 (#10)**	*Dating expectations (L25)*	84.4	49.2 (#17)	58.4 (#15)	53.8 (#17)
*Work at place of education (L21)*	93.3	51.3 (#15)	55.0 (#20)	53.1 (#18)	*Home Support Services (L19)*	83.7	**54.** **6 (#10)**	**65.** **3 (#4)**	**60.** **0 (#7)**
*Fitness/exercise (L4)*	92.6	**62.4 (#3)**	**67.** **5 (#2)**	**65.** **0 (#2)**	*Adulthood expectations (L28)*	83.4	51.3 (#14)	61.5 (#11)	56.4 (#13)
*Needed by others (L11)*	92.1	42.2 (#26)	45.8 (#26)	44.0 (#26)	*Sexual expectations (L26)*	80.0	49.4 (#16)	**61.** **8 (#10)**	55.6 (#15)
*Assistive technologies (L20)*	89.8	**58.2 (#6)**	**64.** **7 (#5)**	**61.** **4 (#5)**	*General health/feel (L1)*	79.9	53.1 (#12)	**66.** **6 (#3)**	**59.** **8 (#8)**
*Relationship with family members (L7)*	89.8	47.5 (#22)	52.9 (#24)	50.2 (#23)	*Playing/hanging out others (L12)*	79.1	44.9 (#25)	56.8 (#19)	50.9 (#22)
*Participation in community activities (L13)*	88.6	39.8 (#27)	44.9 (#27)	42.4 (#27)	*Parenthood expectations (L27)*	77.1	48.9 (#18)	**63.** **4 (#7)**	56.2 (#14)
*Relationship with friends (L8)*	88.5	48.1 (#21)	54.3 (#21)	51.2 (#21)					
**Health Domains**									
*Pressure injuries (H17)*	**122.** **9**	50.3 (#15)	61.8 (#13)	56.1 (#15)	*Dressing/undressing (H6)*	87.3	**61.** **6 (#9)**	**70.** **5 (#6)**	**66.** **1 (#7)**
*Menstrual periods (H13)*	**122.** **8**	60.2 (#13)	49.0 (#16)	54.6 (#16)	*Breathing/coughing (H3)*	87.3	**61.** **4 (#10)**	**70.** **4 (#7)**	**65.** **9 (#8)**
*Sexual activity (H14)*	**112.** **6**	55.2 (#14)	48.9 (#17)	52.1 (#17)	*Leg/foot movement (H7)*	85.9	**67.** **8 (#1)**	**79.** **0 (#1)**	**73.** **4 (#1)**
*Pain (H15)*	**105.** **6**	61.4 (#11)	59.2 (#15)	60.9 (#11)	*Sit-to-stand (H8)*	85.0	**64.** **5 (#4)**	**75.** **9 (#2)**	**70.** **2 (#2)**
*Spasms (H16)*	**101.** **1**	60.7 (#12)	60.1 (#14)	60.4 (#12)	*Walking/ability to move (H9)*	84.2	**63.** **1 (#7)**	**75.** **0 (#3)**	**69.** **1 (#3)**
*Bowel (H12)*	**100.** **9**	**66.0 (#2)**	65.3 (#12)	65.5 (#10)	*Concentration/learning (H1)*	79.3	53.6 (#16)	**67.** **5 (#10)**	60.7 (#13)
*Transfer movements (H10)*	94.2	**63.8 (#5)**	**67.** **7 (#9)**	**65.** **8 (#9)**	*Ability to command attention (H2)*	75.2	49.5 (#17)	65.8 (#11)	57.6 (#14)
*Bladder (H11)*	93.0	**64.6 (#3)**	**69.** **4 (#8)**	**67.** **1 (#5)**					
*Arm/hand movement (H4)*	87.6	**63.7 (#6)**	**72.** **7 (#4)**	**68.** **2 (#4)**					
*Eating/drinking (H5)*	87.4	**62.0 (#8)**	**70.** **9 (#5)**	**66.** **5 (#6)**					

*Note:* H = health domain; L = life domain. A greater preference of overall priorities by individuals with SCI when compared with their P&C (*) or overall priorities within the top ten for each group (^†^) are presented in bold.

## Data Availability

The original contributions presented in the study are included in the article/Supplementary Material, and further inquiries can be directed to the corresponding author.

## Supplementary Materials

The following supporting information can be downloaded at https://www.mdpi.com/article/10.3390/children11121415/s1, File S1: English version of the *L&HDQ*; File S2: List of life and health domains items of the *L&HDQ*; File S3: Supplemental tables.
