# Supplementary material for: Priorities of the Pediatric Spinal Cord Injury Population: An International Study on Patient-Reported Outcome Measures
Source: Children (Basel). 2024 Nov 23;11(12):1415. doi: 10.3390/children11121415 (PMC11674952; doi:10.3390/children11121415)
Supplement: Supplementary file 1 [file children-11-01415-s001.zip › File S1-English H_LDQ.pdf]

Please do not fill in; for coordinators only.
ID# ________________________
Date:________________________

Part I:

Basic Information Form

English version (UK)

PARENT/CAREGIVER REPORT for **YOUNG PERSONS** (***Up to 17 years***)

DIRECTIONS

For parent/caregiver of youth aged up to 17 years: Please answer these questions about your child (part A: questions 1-10) and, where indicated, yourself (part B: questions 11-12).

Note: A caregiver is the main person who helps a young person with spinal cord injury with his or her physical and/or emotional needs. This can be a parent, an extended family member, a close friend or a paid carer.

Part A: About your child (Up to 17 years)	
1. Current date	_ _/_ _/_ _ _ _ (DD/MM/YYYY)
2. Date of birth	_ _/_ _/_ _ _ _ (DD/MM/YYYY)
3. Sex	☐ Male<br>☐ Female
4. Date of injury / onset spinal cord dysfunction	_ _/_ _/_ _ _ _ (DD/MM/YYYY)
5. Level of spinal cord injury / dysfunction (current situation)	☐ Quadriplegia or tetraplegia (= legs and arms affected)<br>☐ Paraplegia (= legs affected)<br>☐ I am not sure
6. Severity of spinal cord injury / dysfunction (current situation)	☐ Complete injury<br>☐ Incomplete injury<br>☐ I am not sure

8. Cause of injury / spinal cord dysfunction	☐ Motor vehicle/pedestrian accident<br>☐ Violence<br>☐ Sports<br>☐ Fall<br>☐ Other accident: ……………<br>☐ Tumor<br>☐ Inflammation/Infection<br>☐ Transverse myelitis<br>☐ Bleeding<br>☐ Surgical complication<br>☐ Other: ….
9. Current education or job (select all that apply)	☐ Nursery school<br>☐ Primary school<br>☐ Secondary school<br>☐ Vocational training/Apprenticeship<br>☐ College<br>☐ Undergraduate university degree<br>☐ Postgraduate university degree<br>☐ Employed: …………………… (JOB)<br>☐ Not attending school, not working<br>☐ Other: ……………………
10. If not attending school anymore: highest completed education	☐ Primary school<br>☐ Secondary school<br>☐ Vocational training/Apprenticeship<br>☐ College<br>☐ Undergraduate university degree<br>☐ Postgraduate university degree<br>☐ Other: ……………………

Part B: About you, the caregiver (*for those caring for young persons aged up to 17 years*)	
11. Highest completed education; caregiver 1	☐ Primary school<br>☐ Secondary school<br>☐ Vocational training/Apprenticeship<br>☐ College<br>☐ Undergraduate university degree<br>☐ Postgraduate university degree<br>☐ Other: ……………………<br>☐ Do not know
12. Highest completed education; caregiver 2<br>(where applicable)	☐ Primary school<br>☐ Secondary school<br>☐ Vocational training/Apprenticeship<br>☐ College<br>☐ Undergraduate university degree<br>☐ Postgraduate university degree<br>☐ Other: ……………………<br>☐ Do not know<br>☐ N/A – there is no caregiver 2

ID# ______________________

Date: ______________________

Part I:

Basic Information Form

English version (UK)

SELF REPORT for **YOUNG ADULTS** (***18-25 years***)

DIRECTIONS

For young adults ages 18-25 years who experienced a spinal cord injury during childhood: Please answer the first set of questions about yourself (part A: questions 1-10) and the second set of questions (part B: questions 11-12) about your parent/caregiver.

Note: A caregiver is the main person who helps a young person with spinal cord injury with his or her physical and/or emotional needs. This can be a parent, an extended family member, a close friend or a paid carer.

**Part A: About yourself (18-25 years)**	
1. Current date	_ _/_ _/_ _ _ _ (DD/MM/YYYY)
2. Date of birth	_ _/_ _/_ _ _ _ (DD/MM/YYYY)
3. Sex	☐ Male<br>☐ Female
4. Ethnicity	White:<br>☐ British<br>☐ Irish<br>☐ Any other White background<br><br>Mixed:<br>☐ White and Black Caribbean<br>☐ White and Black African<br>☐ White and Asian<br>☐ Any other mixed background<br><br>Asian or Asian British:<br>☐ Indian<br>☐ Pakistani<br>☐ Bangladeshi<br>☐ Any other Asian background<br><br>Black or Black British:<br>☐ Caribbean<br>☐ African<br>☐ Any other Black background<br><br>☐ I am not sure<br>☐ Other: ….

5. Date of injury / onset spinal cord dysfunction	_ _/ _ _/ _ _ _ _ (DD/MM/YYYY)
6. Level of spinal cord injury / dysfunction (current situation)	☐ Quadriplegia or tetraplegia (= legs and arms affected)<br>☐ Paraplegia (= legs affected)<br>☐ I am not sure
7. Severity of spinal cord injury / dysfunction (current situation)	☐ Complete injury<br>☐ Incomplete injury<br>☐ I am not sure

8. Cause of injury / spinal cord dysfunction	☐ Motor vehicle/pedestrian accident<br>☐ Violence<br>☐ Sports<br>☐ Fall<br>☐ Other accident: ……………<br>☐ Tumor<br>☐ Inflammation/Infection<br>☐ Transverse myelitis<br>☐ Bleeding<br>☐ Surgical complication<br>☐ Other: ….
9. Current education or job (select all that apply)	☐ Nursery school<br>☐ Primary school<br>☐ Secondary school<br>☐ Vocational training/Apprenticeship<br>☐ College<br>☐ Undergraduate university degree<br>☐ Postgraduate university degree<br>☐ Employed: …………………… (JOB)<br>☐ Not attending school, not working<br>☐ Other: ……………………
10. If not attending school anymore: highest completed education	☐ Primary school<br>☐ Secondary school<br>☐ Vocational training/Apprenticeship<br>☐ College<br>☐ Undergraduate university degree<br>☐ Postgraduate university degree<br>☐ Other: ……………………

**Part B: About the caregiver** (*for those caring for young adults 18-25 years*)	
11. Highest completed education; caregiver 1	☐ Primary school<br>☐ Secondary school<br>☐ Vocational training/Apprenticeship<br>☐ College<br>☐ Undergraduate university degree<br>☐ Postgraduate university degree<br>☐ Other: ……………………<br>☐ Do not know
12. Highest completed education; caregiver 2<br>(where applicable)	☐ Primary school<br>☐ Secondary school<br>☐ Vocational training/Apprenticeship<br>☐ College<br>☐ Undergraduate university degree<br>☐ Postgraduate university degree<br>☐ Other: ……………………<br>☐ Do not know<br>☐ N/A – there is no caregiver 2

Part II: H&LDQ

Health and Life Domain Questionnaire for children with spinal cord injury ages 8-12

English version (UK/US)

DIRECTIONS

On the next pages you will be asked how happy you are with different parts of your life.

Then we're going to ask you some questions about how much you would like your doctor to study and help with problems with your spinal cord injury.

Please think about your feelings over the past month while answering these questions and circle the number that is most true for you. There are no right or wrong answers, but please circle only one number for each question.

You can get help from your parents if you do not understand any of questions.

Please remember that you do not need to answer some questions if they make you uncomfortable or embarrassed.

	**How happy or unhappy are you with this part of your life?**<br>1 = Very unhappy<br>2 = Unhappy<br>3 = Neither happy nor unhappy<br>4 = Happy<br>5 = Very happy<br>NA = This does not apply to me
1. How you feel in general	1 2 3 4 5 NA
2. How easy it is for you to do things you need to do	1 2 3 4 5 NA
3. How you feel inside (think about your feelings and emotions)	1 2 3 4 5 NA
4. Your fitness and exercise	1 2 3 4 5 NA
5. How you look	1 2 3 4 5 NA
6. What you do to have fun	1 2 3 4 5 NA
7. Your relationships with family members	1 2 3 4 5 NA
8. Your friendships	1 2 3 4 5 NA
9. Your ability to help others	1 2 3 4 5 NA
10. Your time playing with or hanging out with others	1 2 3 4 5 NA
11. Your participation in community activities	1 2 3 4 5 NA
12. Your ability to get around inside your home	1 2 3 4 5 NA
13. Your ability to get around places in your community (including stores/shops, restaurants, etc.)	1 2 3 4 5 NA
14. Your ability to see doctors or get the medical care you need	1 2 3 4 5 NA
15. How easy it is to get where you need to go (including by car, bus, train)	1 2 3 4 5 NA
16. Support services you receive in your home (including therapy)	1 2 3 4 5 NA
17. Your equipment and assistive technologies	1 2 3 4 5 NA
18. Your ability to learn new things and to concentrate	1 2 3 4 5 NA
19. Your ability to make yourself heard and catch someone’s attention when you want to talk to them	1 2 3 4 5 NA

	How happy or unhappy are you with this part of your life?
	1 = Very unhappy<br>2 = Unhappy<br>3 = Neither happy nor unhappy<br>4 = Happy<br>5 = Very happy<br>NA = This does not apply to me
20. Your school work (think about your classes)	1 2 3 4 5 NA
21. Your moving around the school (think about how easily you can get around the school building(s))	1 2 3 4 5 NA
22. Your friends and classmates at school (think about who you talk to)	1 2 3 4 5 NA
23. Your ability to breath and cough	1 2 3 4 5 NA
24. Your ability to move your arms and hands	1 2 3 4 5 NA
25. Your ability to eat and drink	1 2 3 4 5 NA
26. Your ability to get yourself dressed and undressed	1 2 3 4 5 NA
27. Your ability to get yourself cleaned up in the bath or shower	1 2 3 4 5 NA
28. Your ability to move your legs and feet	1 2 3 4 5 NA
29. Your ability to get out of your chair and stand	1 2 3 4 5 NA
30. Your ability to walk	1 2 3 4 5 NA
31. Your ability to get from one place to another, such as your bed to chair, or chair to toilet	1 2 3 4 5 NA
32. How you empty your bladder (how you wee/pee)	1 2 3 4 5 NA
33. How you empty your bowel (how you poo/poop)	1 2 3 4 5 NA
34. Having pain and how you can make them better	1 2 3 4 5 NA
35. Having spasms, or muscle jumping/cramps, and how you can make them better	1 2 3 4 5 NA
36. Having skin (pressure) sores, or ulcers, and how you can stop them from starting	1 2 3 4 5 NA

**Do you think it is important for your doctor to study this problem?**	1 = Very unimportant<br>2 = Unimportant<br>3 = Neither important nor unimportant<br>4 = Important<br>5 = Very important
1. How you feel physically (your health)	1 2 3 4 5
2. How you feel emotionally (think about your feelings)	1 2 3 4 5
3. Your relationships with others	1 2 3 4 5
4. Your participation in activities (including sports, clubs, and other activities)	1 2 3 4 5
5. Your ability to get around places you go (including at home and in the community)	1 2 3 4 5
6. Your ability to take care of your daily personal needs (like going to the bathroom, getting washed up, and getting dressed)	1 2 3 4 5
7. Your experience at school	1 2 3 4 5

**How much help did you need in completing all of the above questions?**	
I needed help and explanation from someone else to answer the questions	☐ YES<br>☐ NO
I needed help from someone else to circle/mark the answers to the questions	☐ YES<br>☐ NO

PLEASE CHECK WHETHER YOU HAVE ANSWERED AND CIRCLED ALL QUESTIONS ON THE PREVIOUS PAGES

***OPTIONAL**: Would you like your doctor and others to investigate any other issues related to spinal cord injury in kids that were not mentioned on these pages? If so, please fill those in here:*

OTHER ASPECTS	**Do you think it is important for your doctor to study this problem?**<br>1 = Very unimportant<br>2 = Unimportant<br>3 = Neither important nor unimportant<br>4 = Important<br>5 = Very important
O.1.	1 2 3 4 5
O.2.	1 2 3 4 5
O.3.	1 2 3 4 5
O.4.	1 2 3 4 5
O.5.	1 2 3 4 5

Part II: H&LDQ

Health and Life Domain Questionnaire for teens and young adults with spinal cord injury (ages 13-25)

English version (UK/US)

DIRECTIONS

On the following pages we will be asking about different parts, or domains, of your life. These are listed in the first column. We are going to ask three questions for each:

a) How **important** is this domain to your daily life,
b) How **happy** are you with this part of your life, and
c) How much would you like your doctor to study and help with problems with your spinal cord injury.

Please think about your feelings over the past month while answering these questions and circle the number most true for you. You should be circling three answers for each row (or domain), but only one answer within each box.

You can get help from your parents if you do not understand any of the questions.

Please remember that you do not need to answer some questions if they make you uncomfortable or embarrassed.

LIFE DOMAINS	How important or unimportant do you think this domain is to your daily life?	How happy or unhappy are you with this part of your life?	Do you think it is important for your doctor to study this problem?
	1 = Very unimportant<br>2 = Unimportant<br>3 = Neither important, nor unimportant<br>4 = Important<br>5 = Very important<br>NA = Does not apply to me	1 = Very unhappy<br>2 = Unhappy<br>3 = Neither happy, nor unhappy<br>4 = Happy<br>5 = Very happy<br>NA = Does not apply to me	1= Very unimportant<br>2 = Unimportant<br>3 = Neither important, nor unimportant<br>4 = Important<br>5 = Very important
L.1. How you feel in general	1 2 3 4 5 NA	1 2 3 4 5 NA	1 2 3 4 5
L.2. Your physical functioning	1 2 3 4 5 NA	1 2 3 4 5 NA	1 2 3 4 5
L.3. How you feel inside, in terms of your emotions and feelings	1 2 3 4 5 NA	1 2 3 4 5 NA	1 2 3 4 5
L.4. Your fitness and exercise	1 2 3 4 5 NA	1 2 3 4 5 NA	1 2 3 4 5
L.5. How you look	1 2 3 4 5 NA	1 2 3 4 5 NA	1 2 3 4 5
L.6. What you do to have fun	1 2 3 4 5 NA	1 2 3 4 5 NA	1 2 3 4 5
L.7. Your relationships with family members	1 2 3 4 5 NA	1 2 3 4 5 NA	1 2 3 4 5
L.8. Your relationships with friends	1 2 3 4 5 NA	1 2 3 4 5 NA	1 2 3 4 5
L.9. Your communication with others	1 2 3 4 5 NA	1 2 3 4 5 NA	1 2 3 4 5

LIFE DOMAINS	How important or unimportant do you think this domain is to your daily life?						How happy or unhappy are you with this part of your life?						Do you think it is important for your doctor to study this problem?				
	1 = Very unimportant<br>2 = Unimportant<br>3 = Neither important, nor unimportant<br>4 = Important<br>5 = Very important<br>NA = Does not apply to me						1= Very unhappy<br>2 = Unhappy<br>3 = Neither happy, nor unhappy<br>4 = Happy<br>5 = Very happy<br>NA = Does not apply to me						1 = Very unimportant<br>2 = Unimportant<br>3 = Neither important, nor unimportant<br>4 = Important<br>5 = Very important				
L.10. Your ability to help others	1	2	3	4	5	NA	1	2	3	4	5	NA	1	2	3	4	5
L.11. How much you are needed by others	1	2	3	4	5	NA	1	2	3	4	5	NA	1	2	3	4	5
L.12. Your time playing with or hanging out with others	1	2	3	4	5	NA	1	2	3	4	5	NA	1	2	3	4	5
L.13. Your participation in community activities	1	2	3	4	5	NA	1	2	3	4	5	NA	1	2	3	4	5
L.14. Your ability to get around inside your home	1	2	3	4	5	NA	1	2	3	4	5	NA	1	2	3	4	5
L.15. Your ability to get around places in your community (including stores/shops, restaurants, etc.)	1	2	3	4	5	NA	1	2	3	4	5	NA	1	2	3	4	5
L.16. Your ability to see doctors or get medical care	1	2	3	4	5	NA	1	2	3	4	5	NA	1	2	3	4	5

LIFE DOMAINS	How important or unimportant do you think this domain is to your daily life?	How happy or unhappy are you with this part of your life?	Do you think it is important for your doctor to study this problem?
	1 = Very unimportant<br>2 = Unimportant<br>3 = Neither important, nor unimportant<br>4 = Important<br>5 = Very important<br>NA = Does not apply to me	1 = Very unhappy<br>2 = Unhappy<br>3 = Neither happy, nor unhappy<br>4 = Happy<br>5 = Very happy<br>NA = Does not apply to me	1 = Very unimportant<br>2 = Unimportant<br>3 = Neither important, nor unimportant<br>4 = Important<br>5 = Very important
L.17. How easy it is to get where you need to go (including by car, bus, train)	1 2 3 4 5 NA	1 2 3 4 5 NA	1 2 3 4 5
L.18. Your ability to take care of your daily personal needs (including dressing, bathing, and toileting)	1 2 3 4 5 NA	1 2 3 4 5 NA	1 2 3 4 5
L.19. Support services you receive in your home (including therapy)	1 2 3 4 5 NA	1 2 3 4 5 NA	1 2 3 4 5
L.20. Your equipment and assistive technologies	1 2 3 4 5 NA	1 2 3 4 5 NA	1 2 3 4 5
L.21. Your school/college/university, in terms of school/college/university work	1 2 3 4 5 NA	1 2 3 4 5 NA	1 2 3 4 5
L.22. Your school/college/university, in terms of how easily you can get around the school/college/university building(s)	1 2 3 4 5 NA	1 2 3 4 5 NA	1 2 3 4 5

LIFE DOMAINS	**How important or unimportant do you think this domain is to your daily life?**	**How happy or unhappy are you with this part of your life?**	**Do you think it is important for your doctor to study this problem?**
	1 = Very unimportant<br>2 = Unimportant<br>3 = Neither important, nor unimportant<br>4 = Important<br>5 = Very important<br>NA = Does not apply to me	1= Very unhappy<br>2 = Unhappy<br>3 = Neither happy, nor unhappy<br>4 = Happy<br>5 = Very happy<br>NA = Does not apply to me	1 = Very unimportant<br>2 = Unimportant<br>3 = Neither important, nor unimportant<br>4 = Important<br>5 = Very important
L.23. Your school/college/university, in terms of peer (social) relationships	1 2 3 4 5 NA	1 2 3 4 5 NA	1 2 3 4 5
L.24. Your current situation, and/or future expectations, regarding employment	1 2 3 4 5 NA	1 2 3 4 5 NA	1 2 3 4 5
L.25. Your current situation, and/or future expectations, regarding dating	1 2 3 4 5 NA	1 2 3 4 5 NA	1 2 3 4 5
L.26. Your current situation, and/or future expectations, regarding sexual relationships	1 2 3 4 5 NA	1 2 3 4 5 NA	1 2 3 4 5
L.27. Your expectations regarding having children and being a parent	1 2 3 4 5 NA	1 2 3 4 5 NA	1 2 3 4 5
L.28. Your readiness and expectations regarding your transition to adulthood	1 2 3 4 5 NA	1 2 3 4 5 NA	1 2 3 4 5

HEALTH DOMAINS	How important or unimportant do you think this domain is to your daily life?	How happy or unhappy are you with this part of your life?	Do you think it is important for your doctor to study this problem?
	1 = Very unimportant<br>2= Unimportant<br>3 = Neither important, nor unimportant<br>4 = Important<br>5 = Very important<br>NA = Does not apply to me	1 = Very unhappy<br>2 = Unhappy<br>3 = Neither happy, nor unhappy<br>4 = Happy<br>5 = Very happy<br>NA = Does not apply to me	1= Very unimportant<br>2 = Unimportant<br>3 = Neither important, nor unimportant<br>4 = Important<br>5 = Very important
H.1. Your ability to learn new things and to concentrate	1 2 3 4 5 NA	1 2 3 4 5 NA	1 2 3 4 5
H.2. Your ability to make yourself heard and catch someone's attention	1 2 3 4 5 NA	1 2 3 4 5 NA	1 2 3 4 5
H.3. Your ability to breath and cough	1 2 3 4 5 NA	1 2 3 4 5 NA	1 2 3 4 5
H.4. Your ability to move your arms and hands	1 2 3 4 5 NA	1 2 3 4 5 NA	1 2 3 4 5
H.5. Your ability to eat and drink	1 2 3 4 5 NA	1 2 3 4 5 NA	1 2 3 4 5
H.6. Your ability to get yourself dressed and undressed	1 2 3 4 5 NA	1 2 3 4 5 NA	1 2 3 4 5
H.7. Your ability to move your legs and feet	1 2 3 4 5 NA	1 2 3 4 5 NA	1 2 3 4 5
H.8. Your ability to get out of your chair and stand	1 2 3 4 5 NA	1 2 3 4 5 NA	1 2 3 4 5

HEALTH DOMAINS	How important or unimportant do you think this domain is to your daily life?	How happy or unhappy are you with this part of your life?	Do you think it is important for your doctor to study this problem?
	1 = Very unimportant<br>2 = Unimportant<br>3 = Neither important, nor unimportant<br>4 = Important<br>5 = Very important<br>NA = Does not apply to me	1 = Very unhappy<br>2 = Unhappy<br>3 = Neither happy, nor unhappy<br>4 = Happy<br>5 = Very happy<br>NA = Does not apply to me	1 = Very unimportant<br>2 = Unimportant<br>3 = Neither important, nor unimportant<br>4 = Important<br>5 = Very important
H.9. Your ability to walk/wheel/move	1 2 3 4 5 NA	1 2 3 4 5 NA	1 2 3 4 5
H.10. Your ability to get from one place to another, such as your bed to chair, or chair to toilet	1 2 3 4 5 NA	1 2 3 4 5 NA	1 2 3 4 5
H.11. Emptying your bladder	1 2 3 4 5 NA	1 2 3 4 5 NA	1 2 3 4 5
H.12. Emptying your bowel	1 2 3 4 5 NA	1 2 3 4 5 NA	1 2 3 4 5
H.13. For girls: Your management of menstrual periods	1 2 3 4 5 NA	1 2 3 4 5 NA	1 2 3 4 5
H.14. Your sexual activity	1 2 3 4 5 NA	1 2 3 4 5 NA	1 2 3 4 5
H.15. The presence of pain and how this is treated	1 2 3 4 5 NA	1 2 3 4 5 NA	1 2 3 4 5
H.16. The presence of spasms, or muscle jumping, and how you can control this	1 2 3 4 5 NA	1 2 3 4 5 NA	1 2 3 4 5

HEALTH DOMAINS	How important or unimportant do you think this domain is to your daily life?	How happy or unhappy are you with this part of your life?	Do you think it is important for your doctor to study this problem?
	1 = Very unimportant<br>2 = Unimportant<br>3 = Neither important, nor unimportant<br>4 = Important<br>5 = Very important<br>NA = Does not apply to me	1 = Very unhappy<br>2 = Unhappy<br>3 = Neither happy, nor unhappy<br>4 = Happy<br>5 = Very happy<br>NA = Does not apply to me	1 = Very unimportant<br>2 = Unimportant<br>3 = Neither important, nor unimportant<br>4 = Important<br>5 = Very important
H.17. The presence of skin (pressure) sores, or ulcers, and how you can stop this from starting	1 2 3 4 5 NA	1 2 3 4 5 NA	1 2 3 4 5

How much help did you need in completing all of the above questions?	
I needed help and explanation from someone else to answer the questions	☐ YES ☐ NO
I needed help from someone else to circle/tick the answers to the questions	☐ YES ☐ NO

PLEASE CHECK WHETHER YOU HAVE ANSWERED AND CIRCLED ALL QUESTIONS!

OPTIONAL: *Would you like spinal cord injury researchers to investigate any other aspects related to spinal cord injury in children and adolescents which were not mentioned on previous pages? If so, please fill in and score:*

**OTHER ASPECTS**	**Do you think it is important for your doctor to study this problem?**<br><br>1 = Very unimportant<br>2 = Unimportant<br>3 = Neither important, nor unimportant<br>4 = Important<br>5 = Very important
O.1.	1 2 3 4 5
O.2.	1 2 3 4 5
O.3.	1 2 3 4 5
O.4.	1 2 3 4 5
O.5.	1 2 3 4 5

Part II: H&LDQ

Health and Life Domain Questionnaire for parent/caregiver of children, adolescents and young adults with spinal cord injury (Up to the age of 25)

English version (UK/US)

DIRECTIONS

On the following pages you will be asked about different parts, or domains, of your child's life. These are listed in the first column. We are going to ask three questions for each:

a) How **important** do you think this domain is to your child's daily life,
b) How **happy** are you with this part of your child's life, and
c) How much would you like the treating doctor to study and help with problems with the spinal cord injury of your child.

You should therefore be circling three answers for each row (or domain), but only one answer within each box.

Note: A caregiver or carer is the main person who helps a young person with spinal cord injury with his or her physical and/or emotional needs. This can be a parent, an extended family member, a close friend or a paid carer.

Please remember that you do not need to answer some questions if they make you uncomfortable or embarrassed.

LIFE DOMAINS	How important do you think this domain is to your child's daily life?	How happy are you with this part of your child's life?	Do you think it is important for your child's doctor to study this problem?
	1 = Very unimportant<br>2 = Unimportant<br>3 = Neither important, nor unimportant<br>4 = Important<br>5 = Very important<br>NA = Does not apply to my child	1 = Very unhappy<br>2 = Unhappy<br>3 = Neither happy, nor unhappy<br>4 = Happy<br>5 = Very happy<br>NA = Does not apply to my child	1 = Very unimportant<br>2 = Unimportant<br>3 = Neither important, nor unimportant<br>4 = Important<br>5 = Very important
L.1. Your child's general health	1 2 3 4 5 NA	1 2 3 4 5 NA	1 2 3 4 5
L.2. Your child's physical functioning	1 2 3 4 5 NA	1 2 3 4 5 NA	1 2 3 4 5
L.3. Your child's general mood	1 2 3 4 5 NA	1 2 3 4 5 NA	1 2 3 4 5
L.4. Your child's fitness and exercise	1 2 3 4 5 NA	1 2 3 4 5 NA	1 2 3 4 5
L.5. Your child's leisure and recreation	1 2 3 4 5 NA	1 2 3 4 5 NA	1 2 3 4 5
L.6. Your child's relationships with family members	1 2 3 4 5 NA	1 2 3 4 5 NA	1 2 3 4 5
L.7. Your child's relationships with friends	1 2 3 4 5 NA	1 2 3 4 5 NA	1 2 3 4 5
L.8. Your child's communication with others	1 2 3 4 5 NA	1 2 3 4 5 NA	1 2 3 4 5

LIFE DOMAINS	How important do you think this domain is to your child's daily life?	How happy are you with this part of your child's life?	Do you think it is important for your child's doctor to study this problem?
	1 = Very unimportant<br>2 = Unimportant<br>3 = Neither important, nor unimportant<br>4 = Important<br>5 = Very important<br>NA = Does not apply to my child	1 = Very unhappy<br>2 = Unhappy<br>3 = Neither happy, nor unhappy<br>4 = Happy<br>5 = Very happy<br>NA = Does not apply to my child	1 = Very unimportant<br>2 = Unimportant<br>3 = Neither important, nor unimportant<br>4 = Important<br>5 = Very important
L.9. Your child's ability to help others	1 2 3 4 5 NA	1 2 3 4 5 NA	1 2 3 4 5
L.10. How much your child is needed by others	1 2 3 4 5 NA	1 2 3 4 5 NA	1 2 3 4 5
L.11. Your child's social activities	1 2 3 4 5 NA	1 2 3 4 5 NA	1 2 3 4 5
L.12. Your child's participation in community activities	1 2 3 4 5 NA	1 2 3 4 5 NA	1 2 3 4 5
L.13. The accessibility of your child's home	1 2 3 4 5 NA	1 2 3 4 5 NA	1 2 3 4 5
L.14. The accessibility of your child's community (including stores/shops, restaurants, etc.)	1 2 3 4 5 NA	1 2 3 4 5 NA	1 2 3 4 5
L.15. Your child's access to healthcare services	1 2 3 4 5 NA	1 2 3 4 5 NA	1 2 3 4 5

LIFE DOMAINS	**How important do you think this domain is to your child's daily life?**	**How happy are you with this part of your child's life?**	**Do you think it is important for your child's doctor to study this problem?**
	1 = Very unimportant<br>2 = Unimportant<br>3 = Neither important, nor unimportant<br>4 = Important<br>5 = Very important<br>NA = Does not apply to my child	1 = Very unhappy<br>2 = Unhappy<br>3 = Neither happy, nor unhappy<br>4 = Happy<br>5 = Very happy<br>NA = Does not apply to my child	1 = Very unimportant<br>2 = Unimportant<br>3 = Neither important, nor unimportant<br>4 = Important<br>5 = Very important
L.16. Your child's availability of transportation (including car, bus, train)	1 2 3 4 5 NA	1 2 3 4 5 NA	1 2 3 4 5
L.17. Your child's ability to take care of his/her daily personal needs (including dressing, bathing, and toileting)	1 2 3 4 5 NA	1 2 3 4 5 NA	1 2 3 4 5
L.18. Support services your child receives in your home (including therapy)	1 2 3 4 5 NA	1 2 3 4 5 NA	1 2 3 4 5
L.19. Your child's equipment and assistive technologies	1 2 3 4 5 NA	1 2 3 4 5 NA	1 2 3 4 5
L.20. Your child's education (school/college/university or day care), in terms of school/college/university work	1 2 3 4 5 NA	1 2 3 4 5 NA	1 2 3 4 5

LIFE DOMAINS	How important do you think this domain is to your child's daily life?	How happy are you with this part of your child's life?	Do you think it is important for your child's doctor to study this problem?
	1 = Very unimportant<br>2 = Unimportant<br>3 = Neither important, nor unimportant<br>4 = Important<br>5 = Very important<br>NA = Does not apply to my child	1 = Very unhappy<br>2 = Unhappy<br>3 = Neither happy, nor unhappy<br>4 = Happy<br>5 = Very happy<br>NA = Does not apply to my child	1 = Very unimportant<br>2 = Unimportant<br>3 = Neither important, nor unimportant<br>4 = Important<br>5 = Very important
L.21. Your child's education (school/college/university or day care), in terms of physical environment, including accommodations	1 2 3 4 5 NA	1 2 3 4 5 NA	1 2 3 4 5
L.22. Your child's education (school/college/university or day care), in terms of peer (social) relationships	1 2 3 4 5 NA	1 2 3 4 5 NA	1 2 3 4 5
L.23. Your child's situation, and/or future expectations, regarding employment	1 2 3 4 5 NA	1 2 3 4 5 NA	1 2 3 4 5
L.24. Your child's situation, and/or future expectations, regarding dating	1 2 3 4 5 NA	1 2 3 4 5 NA	1 2 3 4 5
L.25. Your child's situation, and/or future expectations, regarding sexual relationships	1 2 3 4 5 NA	1 2 3 4 5 NA	1 2 3 4 5

LIFE DOMAINS	How important do you think this domain is to your child's daily life?	How happy are you with this part of your child's life?	Do you think it is important for your child's doctor to study this problem?
	1 = Very unimportant<br>2 = Unimportant<br>3 = Neither important, nor unimportant<br>4 = Important<br>5 = Very important<br>NA = Does not apply to my child	1 = Very unhappy<br>2 = Unhappy<br>3 = Neither happy, nor unhappy<br>4 = Happy<br>5 = Very happy<br>NA = Does not apply to my child	1 = Very unimportant<br>2 = Unimportant<br>3 = Neither important, nor unimportant<br>4 = Important<br>5 = Very important
L.26. Your child's readiness, and/or future expectations, regarding having children and being a parent	1 2 3 4 5 NA	1 2 3 4 5 NA	1 2 3 4 5
L.27. Your child's readiness, and/or future expectations, for the transition to adulthood	1 2 3 4 5 NA	1 2 3 4 5 NA	1 2 3 4 5

HEALTH DOMAINS	How important do you think this domain is to your child's daily life?<br>1 = Very unimportant<br>2 = Unimportant<br>3 = Neither important, nor unimportant<br>4 = Important<br>5 = Very important<br>NA = Does not apply to my child	How happy are you with this part of your child's life?<br>1 = Very unhappy<br>2 = Unhappy<br>3 = Neither happy, nor unhappy<br>4 = Happy<br>5 = Very happy<br>NA = Does not apply to my child	Do you think it is important for your child's doctor to study this problem?<br>1 = Very unimportant<br>2 = Unimportant<br>3 = Neither important, nor unimportant<br>4 = Important<br>5 = Very important
H.1. Your child's ability to concentrate and learn new things	1 2 3 4 5 NA	1 2 3 4 5 NA	1 2 3 4 5
H.2. Your child's ability to make him/herself heard and catch someone's attention	1 2 3 4 5 NA	1 2 3 4 5 NA	1 2 3 4 5
H.3. Your child's ability to breath and cough	1 2 3 4 5 NA	1 2 3 4 5 NA	1 2 3 4 5
H.4. Your child's ability to move his/her arms and hands	1 2 3 4 5 NA	1 2 3 4 5 NA	1 2 3 4 5
H.5. Your child's ability to eat and drink	1 2 3 4 5 NA	1 2 3 4 5 NA	1 2 3 4 5
H.6. Your child's ability to get him/herself dressed and undressed	1 2 3 4 5 NA	1 2 3 4 5 NA	1 2 3 4 5
H.7. Your child's ability to move his/her legs and feet	1 2 3 4 5 NA	1 2 3 4 5 NA	1 2 3 4 5

HEALTH DOMAINS	How important do you think this domain is to your child's daily life?	How happy are you with this part of your child's life?	Do you think it is important for your child's doctor to study this problem?
	1 = Very unimportant<br>2 = Unimportant<br>3 = Neither important, nor unimportant<br>4 = Important<br>5 = Very important<br>NA = Does not apply to my child	1 = Very unhappy<br>2 = Unhappy<br>3 = Neither happy, nor unhappy<br>4 = Happy<br>5 = Very happy<br>NA = Does not apply to my child	1 = Very unimportant<br>2 = Unimportant<br>3 = Neither important, nor unimportant<br>4 = Important<br>5 = Very important
H.8. Your child's ability to get out of his/her chair and stand	1 2 3 4 5 NA	1 2 3 4 5 NA	1 2 3 4 5
H.9. Your child's ability to walk	1 2 3 4 5 NA	1 2 3 4 5 NA	1 2 3 4 5
H. 10. Your child's ability to transfer from one surface to another such as bed to chair, or chair to toilet	1 2 3 4 5 NA	1 2 3 4 5 NA	1 2 3 4 5
H.11. Your child's bladder management	1 2 3 4 5 NA	1 2 3 4 5 NA	1 2 3 4 5
H.12. Your child's bowel management	1 2 3 4 5 NA	1 2 3 4 5 NA	1 2 3 4 5
H.13. For girls: Your child's management of menstrual periods	1 2 3 4 5 NA	1 2 3 4 5 NA	1 2 3 4 5
H.14. Your child's sexual activity	1 2 3 4 5 NA	1 2 3 4 5 NA	1 2 3 4 5

HEALTH DOMAINS	How important do you think this domain is to your child's daily life?	How happy are you with this part of your child's life?	Do you think it is important for your child's doctor to study this problem?
	1 = Very unimportant<br>2 = Unimportant<br>3 = Neither important, nor unimportant<br>4 = Important<br>5 = Very important<br>NA = Does not apply to my child	1 = Very unhappy<br>2 = Unhappy<br>3 = Neither happy, nor unhappy<br>4 = Happy<br>5 = Very happy<br>NA = Does not apply to my child	1 = Very unimportant<br>2 = Unimportant<br>3 = Neither important, nor unimportant<br>4 = Important<br>5 = Very important
H.15. The presence of pain and how this is treated	1 2 3 4 5 NA	1 2 3 4 5 NA	1 2 3 4 5
H.16. The presence of spasms, or muscle jumping, and how your child can control this	1 2 3 4 5 NA	1 2 3 4 5 NA	1 2 3 4 5
H.17. The presence of skin (pressure) sores, or ulcers, and how your child can stop this from starting	1 2 3 4 5 NA	1 2 3 4 5 NA	1 2 3 4 5

OPTIONAL: *Would you like spinal cord injury researchers to investigate any other aspects related to spinal cord injury in children and adolescents which were not mentioned on previous pages? If so, please fill in and score:*

**OTHER ASPECTS**	**Do you think it is important for your child's doctor to study this problem?**<br>1 = Very unimportant<br>2 = Unimportant<br>3 = Neither important, nor unimportant<br>4 = Important<br>5 = Very important
O.1.	1 2 3 4 5
O.2.	1 2 3 4 5
O.3.	1 2 3 4 5
O.4.	1 2 3 4 5
O.5.	1 2 3 4 5

Part III:

Neurology Form

English version (UK)

HEALTHCARE PROFESSIONAL REPORT

DIRECTIONS

On the following page is a list neurological information required for a correct interpretation of survey parts I to III. Please enter relevant data based on the individual's medical record where available.

Note: This form should only be completed once informed consent has been obtained from the participant and his/her caregiver(s).

1. Date of birth	_ _/_ _/_ _ _ _ (DD/MM/YYYY)
2. Gender	☐ Male<br>☐ Female
**At time of onset of spinal cord injury/dysfunction**	
3. Date of assessment	_ _/_ _/_ _ _ _ (DD/MM/YYYY)<br>☐ Do not know
4a. Level of spinal cord injury/dysfunction<br>*If no detailed information is available: go to item 4b. Otherwise please proceed to item 5.*	▪ Motor level of injury (ISNCSCI)<br>Left: _ _ Right: _ _<br>▪ Sensory level of injury (ISNCSCI)<br>Left: _ _ Right: _ _
4b. Level of spinal cord injury/dysfunction	☐ Tetraplegia<br>☐ Paraplegia<br>☐ Cauda Equina<br>☐ Do not know
5. Severity of spinal cord injury/dysfunction	☐ Motor & sensory complete (AIS grade A)<br>☐ Motor complete (AIS grade B)<br>☐ Motor incomplete (AIS grade C,D)<br>☐ Do not know
**Current situation**	
6. Date of assessment	_ _/_ _/_ _ _ _ (DD/MM/YYYY)<br>☐ Do not know
7a. Level of spinal cord injury/dysfunction<br>*If no detailed information is available: go to item 7b. Otherwise please proceed to item 8.*	▪ Motor level of injury (ISNCSCI)<br>Left: _ _ Right: _ _<br>▪ Sensory level of injury (ISNCSCI)<br>Left: _ _ Right: _ _

7b. Level of spinal cord injury/dysfunction	☐ Tetraplegia<br>☐ Paraplegia<br>☐ Cauda Equina<br>☐ Do not know
8. Severity of spinal cord injury/dysfunction	☐ Motor & sensory complete (AIS grade A)<br>☐ Motor complete (AIS grade B)<br>☐ Motor incomplete (AIS grade C,D)<br>☐ Recovered (AIS grade E)<br>☐ Do not know
7. Other comments:	
