# Supplementary material for: Priorities of the Pediatric Spinal Cord Injury Population: An International Study on Patient-Reported Outcome Measures
Source: Children (Basel). 2024 Nov 23;11(12):1415. doi: 10.3390/children11121415 (PMC11674952; doi:10.3390/children11121415)
Supplement: Supplementary file 1 [file children-11-01415-s001.zip › File S2-List H_LDQ.pdf]

Table S1. List of health and life domain items of the H&LDQ for children aged 13-25 years old.

Life domain
L1. How you feel in general.
L2. Your physical functioning.
L3. How you feel inside, in terms of your emotions and feelings.
L4. Your fitness and exercise.
L5. How you look.
L6. What you do to have fun.
L7. Your relationship with family members.
L8. Your relationships with friends.
L9. Your communication with others.
L10. Your ability to help others.
L11. How much you are needed by others.
L12. Your time playing with or hanging out with others.
L13. Your participation in community activities.
L14. Your ability to get around inside your home.
L15. Your ability to get around places in your community (including stores/shops, restaurants, etc.).
L16. Your ability to see doctors or get the medical care.
L17. How easy it is to get where you need to go (including by car, bus, train).
L18. Your ability to take care of your daily personal needs (including dressing, bathing, and toileting).
L19. Support services you receive in your home (including therapy).
L20. Your equipment and assistive technologies.
L21. Your school/college/university, in terms of school/college/university work.
L22. Your school/college/university, in terms of how easily you can get around the school/college/university buildings(s).
L23. Your school/college/university, in terms of peer (social) relationships.
L24. Your current situation, and/or future expectations, regarding employment.
L25. Your current situation, and/or future expectations, regarding dating.
L26. Your current situation and/or future expectations, regarding sexual relationships.
L27. Your expectations regarding having children and being a parent.
L28. Your readiness and expectations regarding your transition to adulthood.

Health domain
H1. Your ability to learn new things and to concentrate.
H2. Your ability to make yourself heard and catch someone's attention.
H3. Your ability to breath and cough.
H4. Your ability to move your arms and hands.
H5. Your ability to eat and drink.
H6. Your ability to get yourself dressed and undressed.
H7. Your ability to move your legs and feet.
H8. Your ability to get out of your chair and stand.
H9. Your ability to walk/wheel/move.
H10. Your ability to get from one place to another, such as your bed to chair, or chair to toilet.
H11. Emptying your bladder.
H12. Emptying your bowel.
H13. For girls: Your management of menstrual periods.
H14. Your sexual activity.
H15. The presence of pain and how this is treated.
H16. The presence of spams, or muscle jumping, and how you can control this.
H17. The presence of skin (pressure) injury, or ulcers, and how you can stop this from starting.

Table S2. List of health and life domain items of the H&LDQ for children aged 8-12 years old.

Life domain: unhappiness
L1. How you feel in general.
L2. How easy it is for you to do things you need to do.
L3. How you feel inside (think about your feelings and emotions).
L4. Your fitness and exercise.
L5. How you look.
L6. What you do to have fun.
L7. Your relationship with family members.
L8. Your friendships.
L9. Your ability to help others.
L10. Your time playing with or hanging out with others.
L11. Your participation in community activities.
L12. Your ability to get around inside your home.
L13. Your ability to get around places in your community (including stores/shops, restaurants, etc.).
L14. Your ability to see doctors or get the medical care you need.
L15. How easy it is to get where you need to go (including by car, bus, train).
L16. Support services you receive in your home (including therapy).
L17. Your equipment and assistive technologies.
L18. Your ability to learn new things and to concentrate.
L19. Your ability to make yourself heard and catch someone's attention when you want to talk to them.
L20. Your school work (think about your classes).
L21. Your moving around the school (think about how easily you can get around the school building (s)).
L22. Your friends and classmates at school (think about who you talk to).

Life domain: research
L23. How you feel physically (your health).
L24. How you feel emotionally (think about your feelings).
L25. Your relationship with others.
L26. Your participation in activities (including sports, clubs, and other activities).
L27. Your ability to get around places you go (including at home and in the community).
L28. Your ability to take care of your daily personal needs (like going to the bathroom, getting washed up, and getting dressed).
L29. Your experience at school.

Health domain: unhappiness
H1. Your ability to breath and cough.
H2. Your ability to move your arms and hands.
H3. Your ability to eat and drink.
H4. Your ability to get yourself dressed and undressed.
H5. Your ability to get yourself cleaned up in the bath or shower.
H6. Your ability to move your legs and feet.
H7. Your ability to get out of your chair and stand.
H8. Your ability to walk.
H9. Your ability to get from one place to another, such as your bed to chair, or chair to toilet.
H10. How you empty your bladder (how you wee/pee).
H11. How you empty your bowel (how you poo/poop).
H12. Having pain and how you can make them better.
H13. Having spasms, or muscle jumping/cramps, and how you can make them better.
H14. Having skin (pressure) injury, or ulcers and how you can stop them from starting.

Table S3. List of health and life domain items of the H&LDQ for parents and caregivers.

Life domain
L1. Your child's general health.
L2. Your child's physical functioning.
L3. Your child's general mood.
L4. Your child's fitness and exercise.
L5. Your child's leisure and recreation.
L6. Your child's relationship with family members.
L7. Your child's relationships with friends.
L8. Your child's communication with others.
L9. Your child's ability to help others.
L10. How much your child is needed by others.
L11. Your child's social activities.
L12. Your child's participation in community activities.
L13. The accessibility of your child's home.
L14. The accessibility of your child's community (including stores/shops, restaurants, etc.).
L15. Your child's access to healthcare services.
L16. Your child's availability of transportation (including car, bus, train).
L17. Your child's ability to take care of his/her daily personal needs (including dressing, bathing, and toileting).
L18. Support services your child receives in your home (including therapy).
L19. Your child's equipment and assistive technologies.
L20. Your child's education (school/college/university or day care), in terms of school/college/university work.
L21. Your child's education (school/college/university or day care), in terms of physical environment, including accommodations.
L22. Your child's education (school/college/university or day care), in terms of peer (social) relationships.
L23. Your child's situation, and/or future expectations, regarding employment.
L24. Your child's situation, and/or future expectations, regarding dating.
L25. Your child's situation and/or future expectations, regarding sexual relationships.
L26. Your child's readiness, and/or future expectations, regarding having children and being a parent.
L27. Your child's readiness, and/or future expectations, for the transition to adulthood.

Health domain
H1. Your child's ability to concentrate and learn new things.
H2. Your child's ability to make him/herself heard and catch someone's attention.
H3. Your child's ability to breath and cough.
H4. Your child's ability to move his/her arms and hands.
H5. Your child's ability to eat and drink.
H6. Your child's ability to get him/herself dressed and undressed.
H7. Your child's ability to move his/her legs and feet.
H8. Your child's ability to get out of his/her chair and stand.
H9. Your ability to walk.
H10. Your child's ability to transfer from one surface to another such as bed to chair, or chair to toilet.
H11. Your child's bladder management.
H12. Your child's bowel management.
H13. For girls: Your child's management of menstrual periods.
H14. Your child's sexual activity.
H15. The presence of pain and how this is treated.
H16. The presence of spams, or muscle jumping, and how your child can control this.
H17. The presence of skin (pressure) injury, or ulcers, and how your child can stop this from starting.
