# Supplementary material for: Priorities of the Pediatric Spinal Cord Injury Population: An International Study on Patient-Reported Outcome Measures
Source: Children (Basel). 2024 Nov 23;11(12):1415. doi: 10.3390/children11121415 (PMC11674952; doi:10.3390/children11121415)
Supplement: Supplementary file 1 [file children-11-01415-s001.zip › File S3-Supplementary C_Supplemental tables.pdf]

Table S4. Ranked Top 10 unhappiness priorities for life and health domains reported by participants with SCI aged 8-12 years and parents and caregivers (n=31), on the basis of the percentage of important (4) and very important (5) scores.

PARTICIPANTS WITH SCI AGED 8-12 YEARS [¥]				PARENTS AND CAREGIVERS OF PARTICIPANTS WITH SCI AGED 8-12 YEARS			
	% 4 and 5 scores	% 5 scores	Median score (IQR)		% 4 and 5 scores	% 5 scores	Median score (IQR)
Pressure injuries (H14)	61 (#1)	11	4 (3-4)	*Leg/foot movement (H7)*	48 (#1)	13	4 (2-4)
Sit-to-stand (H7)	50 (#2)	32	3.5 (1-5)	*Spasms (H16)*	44 (#2)	19	3 (2-4)
Bowel (H11)	50 (#3)	19	3.5 (2-4)	*Bladder (H11)*	40 (#3)	5	2 (1.5-4)
Mobility in the school (L21)	43 (#4)	25	3 (1.7-4.2)	*Dressing/undressing (H6)*	33 (#4)	10	2 (1.7-4)
Leg/foot movement (H6)	42 (#5)	19	3 (2-4)	*Bowel (H12)*	32 (#5)	0	2 (1.2-4)
Bathing (H5)	42 (#6)	19	3 (1.2-4)	*Pressure injuries (H17)*	29 (#6)	17	2.5 (1.7-4)
Ease of arrival to destination (L15)	40 (#7)	23	3 (1.2-4)	*Walking/ ability to move (H9)*	28 (#7)	14	2 (2-4)
Assistive technologies (L17)	39 (#8)	22	3 (2-4)	*Menstrual period management (H13)*	22 (#8)	11	3 (2-3.5)
Bladder (H10)	37 (#9)	19	3 (1.5-4)	*Sexual activity (H14)*	22 (#9)	11	2.5 (1-3)
Spasms (H14)	35 (#10)	10	3 (2.7-4)	*Pain (H15)*	22 (#10)	4	3 (2-3)

SCI: Spinal cord injury. IQR: interquartile range (25th-75th percentiles). [¥] Children (8-12 years) with motor complete injury showed significant greater unhappiness related to “bathing” (p=0.02), “sit-to-stand” (p=0.02), “bowel” function (p=0.01), and “pressure injuries” (p=0.02) items compared to youth with motor incomplete injury. No statistically significant differences were identified between participants with tetraplegia and those with paraplegia for the unhappiness items in 8-12-year-olds (p>0.05). No significant differences in gender or time since injury were found (p>0.05).

Table S5. Ranked Top 10 unhappiness priorities for life and health domains reported by participants with SCI aged 13-25 years and parents and caregivers (n=70), on the basis of the percentage of unhappy (4) and very unhappy (5) scores.

PARTICIPANTS WITH SCI AGED 13-25 YEARS [¥]				PARENTS AND CAREGIVERS OF PARTICIPANTS WITH SCI AGED 13-25 YEARS			
	% 4 and 5 scores	% 5 scores	Median score (IQR)		% 4 and 5 scores	% 5 scores	Median score (IQR)
**LIFE DOMAINS**							
Mobility in the community (L15)	28 (#1)	5	2 (2-4)	***Home Support Services (L18)****	33 (#1)	9	3 (2.5-3)
Fitness/exercise (L4)	25 (#2)	6	3 (2-3)	***Physical function (L2)****	29 (#2)	7	3 (2-3)
Physical function (L2)	23 (#3)	4	2 (2-3)	***Parenthood expectations (L26)****	26 (#3)	3	3 (2-3)
Dating expectations (L25)	22 (#4)	6	3 (2-3)	*Community accessibility (L14)*	26 (#4)	8	2 (2-2.5)
Ease of arrival to destination (L17)	21 (#5)	6	2 (1.7-3)	***Sexual expectations (L25)****	24 (#5)	4	3 (2-3)
Personal needs (L18)	21 (#6)	4	2 (1-3)	*Assistive technologies (L19)*	23 (#6)	6	2 (1.7-3)
Sexual expectations (L26)	18 (#7)	11	3 (2-3)	***Dating expectations (L24)****	22 (#7)	6	3 (2-3)
Parenthood expectations (L27)	17 (#8)	7	3 (2-3)	***Fitness/exercise (L4)****	21 (#8)	4	2 (2-3)
Appearance (L5)	16 (#9)	7	2 (2-3)	*Employment (L23)*	20 (#9)	3	3 (2-3)
Home Support Services (L19)	15 (#10)	5	2 (1-3)	*Adulthood expectations (27)*	19 (#10)	3	2 (2-3)
**HEALTH DOMAINS**							
Leg/foot movement (H7)	26 (#1)	11	2 (1-4)	***Leg/foot movement (H7)****	56 (#1)	33	3 (2-4)
Sit-to-stand (H8)	26 (#2)	15	2 (1-3.7)	***Sit-to-stand (H8)****	54 (#2)	33	3 (2-4)
Sexual activity (H14)	24 (#3)	10	3 (2-3)	***Walking/ ability to move (H9)****	48 (#3)	35	3 (2-5)
Pain (H15)	21 (#4)	5	2 (1-3)	*Eating/drinking (H5)*	38 (#4)	26	1 (1-2)
Transfer movements (H10)	20 (#5)	7	1 (1-2)	***Dressing/undressing (H6)****	38 (#5)	21	2 (1-3)
Bowel (H12)	19 (#6)	6	2 (1-3)	***Arm/hand movement (H4)****	37 (#6)	23	1 (1-2)
Pressure injuries (H17)	19 (#7)	4	2 (1-3)	***Transfer movements (H10)****	36 (#7)	18	2 (1-3)
Walking/ability to move (H9)	17 (#8)	6	2 (1-3)	***Bladder (H11)****	34 (#8)	19	2.5 (2-3.2)
Bladder (H11)	17 (#9)	5	2 (1-3)	***Bowel (H12)****	32 (#9)	18	3 (2-4)
Dressing/undressing (H6)	15 (#10)	3	1 (1-2)	*Concentration/learning (H1)*	31 (#10)	16	2 (1-2)

Note: Grey shading indicates the top 10 health and life domain unhappiness priorities for participants with SCI aged 13-25 years or parents and caregivers of participants with SCI aged 13-25 years, respectively.

SCI: Spinal cord injury. IQR: interquartile range (25th-75th percentiles). *Top 10 unhappiness priorities for life and health domains reported by both participants with SCI and parents or caregivers. [¥] Adolescents and young adults (13-25 years) with tetraplegia reported significantly greater unhappiness related to the "personal needs" (p=0.03), "sexual expectations" (p=0.04), and "transfer movements" (p=0.005) items compared to

youth with paraplegia, while participants with motor complete injury reported higher unhappiness scores than those with motor incomplete injury in “sexual expectations” and “sexual activity” items (p=0.02). Additionally, individuals with SCI less than 5 years evolution reported higher unhappiness scores than those with SCI more than 5 years evolution in “leg/foot movement” item (p=0.03). Girls showed higher unhappiness scores than boys in “ease of arrival to destination” item (p=0.01).
